# Antifibrotic function of cGKI in the kidney

**DOI:** 10.1186/2050-6511-16-S1-A23

**Published:** 2015-09-02

**Authors:** Jens Schlossmann, Elisabeth Schinner, Veronika Wetzl, Lothar Faerber, Franz Hofmann

**Affiliations:** 1 Pharmacology and Toxicology, Institute of Pharmacy, University Regensburg, Germany; 2 Novartis Pharma GmbH, Nürnberg, Germany; 3 Carvas, Technical University Munich, Germany

## Background

Renal fibrosis is an important process in the induction of chronic kidney diseases. Signalling by cGMP was previously shown to suppress fibrotic diseases of the kidney. Therefore, we analysed whether cGMP-dependent protein kinases, namely cGKI, exert antifibrotic function via cGMP.

## Results

The cGKIα isozyme is expressed in the renal medullary interstitium. Unilateral ureter kidney was taken as model for the analysis of interstitial kidney fibrosis in wild type, cGKI-KO and cGKIα rescue mice (which express cGKIα solely in smooth muscle in a cGKI-KO background). We tested whether the pharmacological stimulation of the cGMP/cGKI signalling pathway affects the induction of interstitial kidney fibrosis. For this purpose we used a) NO-independent sGC stimulators (YC-1, Bay41-8543) and b) the pregnancy hormone serelaxin which was shown to induce cGMP levels and is currently tested for treatment of acute heart failure. sGC stimulators effectively suppressed TGFβ levels, myofibroblast differentiation (SMA) and deposition of extracellular matrix (ECM) (collagen, fibronectin) in wild type mice involving RhoA/ROCK signalling in contrast to cGKI-KO and/or cGKIα rescue mice. Serelaxin treatment by osmotic pumps continuously enhanced cGMP concentrations in the kidney. Serelaxin also strongly reduced interstitial kidney fibrosis via diminished cytokines (TGFβ, CTGF), myofibroblasts and ECM and by regulation of matrix metalloproteases (MMP-2, MMP-9) dependent on the presence of cGKI. However, our results indicated that serelaxin might exert different signalling pathways e.g. via MAPK.

## Conclusion

Our results suggest that pharmacological treatment with sGC stimulators or serelaxin enhancing cGMP suppresses interstitial kidney fibrosis via cGMP/cGKI signalling.

